# The varied distribution and impact of *RAS* codon and other key DNA alterations across the translocation cyclin D subgroups in multiple myeloma

**DOI:** 10.18632/oncotarget.15718

**Published:** 2017-02-24

**Authors:** Caleb K. Stein, Charlotte Pawlyn, Shweta Chavan, Leo Rasche, Niels Weinhold, Adam Corken, Amy Buros, Pieter Sonneveld, Graham H. Jackson, Ola Landgren, Tariq Mughal, Jie He, Bart Barlogie, P. Leif Bergsagel, Faith E. Davies, Brian A. Walker, Gareth J. Morgan

**Affiliations:** ^1^ The Myeloma Institute, University of Arkansas for Medical Sciences, Little Rock, Arkansas, USA; ^2^ The Institute of Cancer Research, London, UK; ^3^ Eramus University, Rotterdam, NL; ^4^ Department of Haematology, Newcastle University, Newcastle-upon-Tyne, UK; ^5^ Memorial Sloan Kettering Cancer Center, New York, New York, USA; ^6^ FoundationOne Medicine, Cambridge, Massachusetts, USA; ^7^ Tufts Medical Center, Boston, Massachusetts, USA; ^8^ Mayo Clinic, Scottsdale, Arizona, USA

**Keywords:** multiple myeloma, gene expression profiling, mutational analysis, translocation cyclin D (TC)

## Abstract

We examined a set of 805 cases that underwent DNA sequencing using the FoundationOne Heme (F1H) targeted sequencing panel and gene expression profiling. Known and likely variant calls from the mutational data were analyzed for significant associations with gene expression defined translocation cyclin D (TC) molecular subgroups. The spectrum of *KRAS, NRAS*, and BRAF codon mutations varied across subgroups with NRAS mutations at Q61 codon being common in hyperdiploid (HRD) and t(11;14) myeloma while being rare in MMSET and MAF. In addition, the presence of RAS-RAF mutations was inversely associated with NFκB pathway activation in all subgroups excluding MAF. In the MMSET subgroup, cases with low *FGFR3* expression frequently had RAS-RAF mutations. Conditional inference tree analysis determined that mutation and homozygous deletion of *TP53, CDKN2C*, and *RB1* were key prognostic factors associated with adverse outcome in a non-relapse clinical setting. In conclusion, this study highlights the heterogeneity in the distribution and clinical outcomes of *RAS* codon and other mutations in multiple myeloma dependent upon primary molecular subgroup.

## INTRODUCTION

Analysis of myeloma plasma cells using cytogenetics and fluorescence *in-situ* hybridization (FISH) has formed the basis of genetic subgrouping in myeloma (MM) [1–3]. These investigations generated etiological groups based upon either the presence of a translocation into the immunoglobulin heavy chain (*IGH*) locus (40%) or hyperdiploidy (HRD). Further analysis identified the overexpression of a D-group cyclin as a key aberration that is uniformly dysregulated as part of a convergent evolutionary pathway integrating all of the genetic events leading to MM [4, 5]. In this respect, translocations into the 11q13 locus directly deregulate *CCND1*; 6p21 *CCND3*; and 4p16 (MMSET), 16q23 (MAF), and 20q12 (MAFB) indirectly deregulate *CCND2*. In contrast, the mechanistic basis for cyclin-D dysregulation in HRD myeloma is less clear [6].

Based on these genetic data, a MM classification incorporating the presence of translocations and D-group cyclin dysregulation, the Translocation Cyclin-D classification (TC) [5] was proposed which recognized eight molecular subgroups. Alternative classification models were also proposed with the most widely used of these, the UAMS classification, recognizing seven molecular subgroups [7]. This classification framework was expanded upon by the HOVON group with additional subgroups defined by secondary features, e.g. NFκB activation and *PRL3* expression [8].

Recently, next generation sequencing data has become available, which promises to further refine classification strategies. The spectrum of mutations in MM has been shown to be dominated by mutations in the RAS (43% of patients) and NFκB pathways (17%), and recurrent alterations in genes, including *TP53*, *ATM*, and *ATR*, and secondary translocations into MYC (8q24) that have strong associations with adverse risk [9]. It has also been shown that bi-allelic events in tumor suppressor genes, especially *TP53*, are associated with proliferation and high risk features which lead to progressive disease [10]. In this work, we investigate the associations between DNA mutation and GEP defined molecular subgroups of MM from a large data set with assessment by gene expression and targeted mutational panels (referred to as FoundationOne Heme, or F1H, below) with particular interest in *RAS* codon mutations.

## RESULTS

### Updated TC algorithm

We classify cases throughout according to an updated TC algorithm (TC-6) designed to accurately define molecular subgroups from gene expression data normalized by GCRMA. A detailed description of our methodology is found in the methods section below. This updated TC classification (TC-6) identifies six major subtypes (D1-HRD, D2, CCND1-11q13, CCND3-6p21, MMSET, and MAF) and validated with ≥ 97.9% agreement across iFISH determined translocations on the MRC-IX data for each of t(4;14), t(11;14), and t(14;16) or t(14;20) (Supplementary Table 1). An ordered bar plot illustrates the distinct expression patterns of primary and secondary genes that discriminate subtypes (Supplementary Figure 1). Key copy number abnormalities and GEP70 high risk (HR) are differentially distributed across subgroups, e.g. 1q+, 13q-, and GEP70 HR are enriched in MAF and MMSET subgroups (Table 1 and Supplementary Figure 2A).

**Table 1:** Clinical and biological features of TC-6 subgroups

	Count	Key Up-Regulated Genes	Key Down-Regulated Genes	Primary Translocation	UAMS Molecular Subgroups	1q+	1p-	13q-	17p-	GEP70 HR	Five-year OS
**D1** **HRD**	299 (33.1)	ISL2 TNFSF10 SULF2	CCND2 S100A4 NES	None	HY (84.6) PR (10.0)	29/193 (15.0)	39/193 (20.2)	37/137 (27.0)	11/145 (7.6)	28/299 (9.4)	74.9
**D2**	236 (26.2)	CCND2 PTP4A3	CCND1 DUSP6	None	LB (46.6) PR (31.4)	73/148 (49.3)	27/148 (18.2)	52/101 (51.5)	7/105 (6.7)	30/236 (12.7)	72.9
**CCND1** **11q13**	165 (18.3)	CCND1 SLC8A1	SULF2	t(11;14) CCND1	CD-2 (57.0) CD-1 (35.2)	30/118 (25.4)	10/118 (8.5)	33/92 (35.9)	13/93 (14.0)	11/165 (6.7)	72.1
**CCND3** **6p21**	17 (1.9)	CCND3 USP49		t(6;14) CCND3	CD-2 (52.9) CD-1 (23.5)	2/9 (22.2)	0/9 (0.0)	3/5 (60.0)	0/7 (0.0)	1/17 (5.9)	82.4
**MMSET**	128 (14.2)	CCND2 WHSC1 FGFR3		t(4;14) MMSET	MS (94.5) PR (5.5)	46/77 (59.7)	19/77 (24.7)	40/56 (71.4)	5/55 (9.1)	33/128 (25.8)	60.2
**MAF**	57 (6.3)	CCND2 MAF or MAFB NUAK1	NCAM1	t(14;16) MAF t(14;20) MAFB t(8:14) MAFA	MF (96.5)	28/37 (75.7)	7/37 (18.9)	20/31 (64.5)	5/33 (15.2)	26/57 (45.6)	52.6
**Total**	902 (100.0)					208/585 (35.6)	102/585 (17.4)	185/424 (43.6)	41/439 (9.3)	129/902 (14.3)	70.5

Each TC-6 subgroup has a distinct biology that is reflected in varied distributions of copy number abnormalities (iFISH), GEP70 risk, and overall survival.

Subgroups with over-expression of *CCND2* (D2, MMSET, and MAF) had higher rates of 1q+ and 13q-. MAF and MMSET cases have the highest proportions of GEP70 HR and the poorest outcome.

### Most frequently altered genes stratified by disease stage

Across our data set of cases with paired gene expression and FoundationOne mutational panels, we observed that *NRAS*, *KRAS*, and *TP53* were the most commonly mutated genes. In total, 38.6% of all cases had a RAS-RAF mutation (*KRAS* alone 16.3%, *NRAS* alone 18.3%, *BRAF* alone 3.0%, with co-occurrence in 1.1%) and 11.3% had a mutation or deletion in *TP53* (Table 2). The rate of *TP53* mutation in our data set is elevated when compared to prior studies [9] because of the heterogeneous disease stage, including non-baseline entries, of samples within our data set. Although heterogeneity in disease stage is a potential confounder of subsequent analyses (which we account for by verifying the significance of all main findings in multivariate analyses that include disease stage as a covariate), it also allows us to illustrate directly the association between specific gene alterations and progressive disease. For example, we found that in addition to *TP53*, *CDKN2C* and *RB1* alterations were also significantly, or nearly significantly in the case of *RB1*, associated with progressive disease stage implicating these alterations as key markers of late stage disease (*p-value* < 0.001, 0.056 and 0.025 for *TP53*, *RB1*, and *CDKN2C*, respectively). We also observed that RAS-RAF mutations, especially *NRAS* mutations of the Q61 codon, were more common at or near relapse than at prior disease stages (20.6% of relapse cases have Q61 *NRAS* mutation, 12.7% in prior disease stages: *p-value* = 0.005). None of the remaining key gene alterations were significantly associated with disease stage including mutations previously found to be associated with outcome, e.g. ATM/ATR [9].

**Table 2:** RAS-RAF codon and other key alterations by disease stage

Gene	Total Mutations	Total Deletions	Untreated (*n* = 182)	In Treatment(*n* = 329)	At or Near Relapse(*n* = 294)	Cochran-Armitage Trend Test*p*-value	Relapse vs Prior Stage*p*-value
*RAS-RAF*	311(38.6%)		**57 (31.3%)**	**117 (35.6%)**	**137 (46.6%)**	**< 0.001**	**0.001**
*KRAS*	134 (16.6%)		25 (13.7%)	55 (16.7%)	54 (18.4%)	0.195	0.370
*G12/13*	66 (8.2%)		15 (8.2%)	28 (8.5%)	23 (7.8%)	0.841	0.872
*Q61*	68 (8.4%)		10 (5.5%)	27 (8.2%)	31 (10.5%)	0.053	0.136
*NRAS*	151 (18.8%)		**26 (14.3%)**	**53 (16.1%)**	**72 (24.5%)**	**0.003**	**0.002**
*G12/13*	26 (3.2%)		3 (1.6%)	11 (3.3%)	12 (4.1%)	0.156	0.407
*Q61*	125 (15.5%)		**23 (12.6%)**	**42 (12.8%)**	**60 (20.4%)**	**0.012**	**0.005**
*BRAF V600E*	26 (3.2%)		6 (3.3%)	9 (2.7%)	11 (3.7%)	0.716	0.678
*TP53*	91 (11.3%)	3 (0.4%)	**12 (6.6%)**	**32 (9.7%)**	**50 (17.0%)**	**< 0.001**	**0.001**
*TRAF3*	27 (3.4%)	14 (1.7%)	6 (3.3%)	19 (5.8%)	16 (5.4%)	0.363	0.861
*FGFR3*	25 (3.1%)		3 (1.6%)	11 (3.3%)	11 (3.7%)	0.225	0.563
*RB1*	23 (2.9%)	15 (1.9%)	5 (2.7%)	14 (4.3%)	19 (6.5%)	0.056	0.111
*CDKN2C*	6 (0.7%)	23 (2.9%)	3 (1.6%)	10 (3.0%)	16 (5.4%)	0.025	0.054
*DNMT3A*	26 (3.2%)		3 (1.6%)	13 (4.0%)	10 (3.4%)	0.373	0.999
*ATM/ATR*	32 (4.0%)		2 (1.1%)	20 (6.1%)	10 (3.4%)	0.398	0.657
*TET2*	23 (2.9%)	1 (0.1%)	4 (2.2%)	12 (3.6%)	8 (2.7%)	0.856	0.909
*BIRC3*	2 (0.2%)	21 (2.6%)	6 (3.3%)	8 (2.4%)	9 (3.1%)	0.955	0.965

Across the heterogeneous disease stages present in our data set, we observed that RAS-RAF mutations were more common at relapse than at prior disease stage, primarily due to increase of *NRAS* mutations of Q61 at relapse. In addition, alterations of *TP53* are more common at relapse than prior disease stages, and mutation and deletion of *RB1* and *CDKN2C* are significantly or near significantly associated with progressive disease stage.

All rows with either *p*-value < 0.01 are bolded. Two *p*-values are reported: a Cochran-Armitage trend test that examines progressive change in distribution across three disease stages and relapse vs prior disease stage Fisher exact test. Mutations and deletions are reported in first two columns, and their combined sum (alterations) is divided across disease stages. For cases with co-occurrence of RAS-RAF mutations, the mutation with highest variant allele frequency is reported here.

### Differential distribution of RAS codon and other mutations across TC subgroups

**Figure 1:**
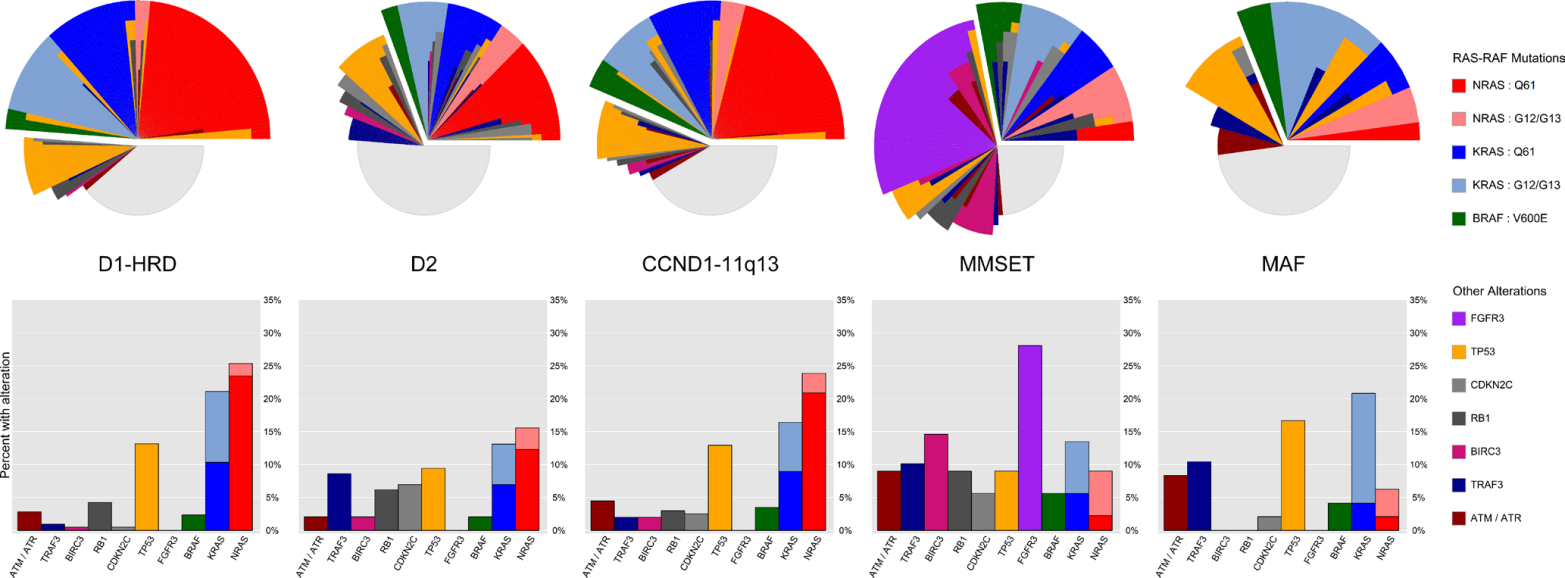
Distribution of RAS-RAF codon and other key alterations across TC-6 subgroups *NRAS* mutations at Q61 are commonly seen in D1-HRD, D2, and CCND1-11q13 subgroups but rare among the MMSET and MAF subtypes. *FGFR3* mutations are exclusive to the MMSET subtype and other genes are altered at a higher frequency in particular subgroups, e.g. MMSET cases are enriched for *BIRC3* alterations and *CCND2* expressing subgroups (D2, MMSET, and MAF) are enriched for *TRAF3* alterations. Stacked bar plots represent frequencies of key alterations reported as percentages within each TC-6 subgroup. For the small number of cases (1.1%) with co-occurrence of RAS-RAF mutations, only the mutation with highest variant allele frequency (VAF) was used. For the radial alteration plots, each individual slice corresponds to the mutational profile of one specific subject.

The distribution of RAS codon mutations is not uniform across TC subgroups. Most notably, *NRAS* mutations, especially at Q61, are common in HRD and t(11;14) MM yet rare in MAF and MMSET (18.5% of D1-HRD (23.5%), D2 (12.3%), and CCND1-11q13 (20.9%) with Q61 *NRAS* vs 2.2% in MAF (2.1%) and MMSET (2.2%): *p-value* < 0.001; Table 3 and Figure 1). This differential pattern of Q61 *NRAS* mutations is independent of disease stage (subgroup-associated *p*-values remain highly significant in multivariate model including disease stage). The rarity of Q61 *NRAS* mutations in MMSET and MAF leads to a proportionally increased rate of *KRAS* and *BRAF* mutations in these subgroups (68% of MMSET and 80% of MAF cases with RAS-RAF mutations are *KRAS* or *BRAF* compared to 48% in D1-HRD, D2, and CCND1-11q13: *p-value* = 0.004). In addition, subgroups with elevated *CCND2* expression (D2, MMSET, and MAF) have fewer RAS-RAF mutations overall compared to cases with elevated *CCND1* expression (D1-HRD, CCND1-11q13) (30% of *CCND2* high expressers with RAS-RAF mutation, 46% of *CCND1* high: *p-value* < 0.001).

**Table 3:** RAS-RAF codon and other key alterations by TC-6 subgroup

Gene	Total Mutations	Total Deletions	Most Common Subgroups	Count (Percentage)	Least Common Subgroups	Count (Percentage)	Fisher Exact Test*p*-value
*RAS-RAF*	311 (38.6%)		**D1-HRD, CCND1**	**192/414 (46.4%)**	**D2, MMSET, MAF**	**115/381 (30.2%)**	**< 0.001**
*KRAS*	134 (16.6%)		D1-HRD, MAF	55/261 (21.1%)	D2, MMSET, CCND1	77/534 (14.4%)	0.020
*G12/13*	66 (8.2%)		MAF	7/48(14.6%)	D1-HRD, D2, CCND1, MMSET	58/747 (7.8%)	0.162
*Q61*	68 (8.4%)		D1-HRD, D2, CCND1	59/658 (9.0%)	MMSET, MAF	8/137(5.8%)	0.309
*NRAS*	151 (18.8%)		**D1-HRD, D2, CCND1**	**140/658 (21.3%)**	**MMSET, MAF**	**11/137 (8.0%)**	**< 0.001**
*G12/13*	26 (3.2%)		MMSET, MAF	8/137(5.8%)	D1-HRD, D2, CCND1	18/658 (2.7%)	0.107
*Q61*	125 (15.5%)		**D1-HRD, D2, CCND1**	**122/658 (18.5%)**	**MMSET, MAF**	**3/137(2.2%)**	**< 0.001**
*BRAF V600E*	26 (3.2%)		MMSET, MAF	7/137(5.1%)	D1-HRD, D2, CCND1	17/658 (2.6%)	0.163
*TP53*	91 (11.3%)	3 (0.4%)	D1-HRD, CCND1, MAF	62/462 (13.4%)	D2, MMSET	31/333 (9.3%)	0.093
*TRAF3*	27 (3.4%)	14 (1.7%)	**D2, MMSET, MAF**	**35/381 (9.2%)**	**D1-HRD, CCND1**	**6/414(1.4%)**	**< 0.001**
*FGFR3*	25 (3.1%)		**MMSET**	**25/89 (28.1%)**	**D1-HRD, D2, CCND1, MAF**	**0/706(0.0%)**	**< 0.001**
*RB1*	23 (2.9%)	15 (1.9%)	D2, MMSET	23/333 (6.9%)	D1-HRD, CCND1, MAF	15/462 (3.2%)	0.019
*CDKN2C*	6 (0.7%)	23 (2.9%)	**D2, MMSET**	**22/333 (6.6%)**	**D1-HRD, CCND1, MAF**	**7/462(1.5%)**	**< 0.001**
*DNMT3A*	26 (3.2%)		D2, CCND1, MMSET	21/534 (3.9%)	D1, MAF	3/261(1.1%)	0.044
*ATM/ATR*	32 (4.0%)		**MMSET, MAF**	**12/137 (8.8%)**	**D1-HRD, D2, CCND1**	**20/658 (3.0%)**	**0.006**
*TET2*	23 (2.9%)	1 (0.1%)	D2, MAF	12/292 (4.1%)	D1-HRD, CCND1, MMSET	11/503 (2.2%)	0.129
*BIRC3*	2 (0.2%)	21 (2.6%)	**MMSET**	**13/89 (14.6%)**	**D1-HRD, D2, CCND1, MAF**	**10/706 (1.4%)**	**< 0.001**

RAS-RAF and other key alterations are not distributed evenly across TC-6 subgroups. We have reported the most significant difference in distribution for each specific gene. A full breakdown of mutation and deletion by subgroup can be found in Supplementary Table 2.

All rows with *p*-values < 0.01 are bolded. Mutations and deletions are reported in first two columns, and their combined sum (alterations) is divided across the TC-6 subgroups. CCND3-6p21 subgroup is not included in this subgroup analysis due to sample size restraints. For cases with co-occurrence of RAS-RAF mutations, the mutation with highest variant allele frequency is reported here.

We also investigated whether specific RAS codon mutations were associated with expression of *CD20* (*MS4A1*) in t(11;14) myeloma as this is a common divisor of this subtype [11, 12]. We found that across all t(11;14) cases, those with RAS-RAF mutations were to some extent less likely to overexpress *CD20* (44.3% with RAS-RAF mutations expressed *CD20*, 60.2% expressed without RAS-RAF mutation; *p-value* = 0.032). This negative association between RAS-RAF mutation and *CD20* expression was found primarily in cases with Q61 *NRAS* mutations as only 33% (14/42) of such cases overexpressed *CD20*. When excluding these cases, we observed no significant difference in the rate of *CD20* overexpression among remaining cases (RAS-RAF mutated excluding Q61 *NRAS* vs non-RAS-RAF mutated cases: *p-value* = 0.708). Thus t(11;14) cases with RAS-RAF mutations appear less likely to overexpress *CD20*, especially those with *NRAS* mutations at Q61.

Overall, we observed an increased frequency in the rate of *NRAS* mutations, especially at Q61, when compared to other cancers. Our data set confirms prior work in MM that identified *NRAS* mutations as more common than *KRAS* and an increased frequency of *NRAS* mutations at Q61 [13]. An enrichment of mutations at Q61 was not observed in cases with *KRAS* mutations (*NRAS*: 83% Q61, 17% G12/G13 compared to *KRAS*: 51% Q61, 49% G12/G13, *p-value* < 0.001; Table 2). While mutations at Q61 compose 60% of *NRAS* mutations in other cancers [14], we find this rate to be over 80% in MM likely due to the increased rate of *NRAS* mutations at Q61 observed in HRD and t(11;14) myeloma—subgroups that comprise the majority of MM.

Other key mutations were enriched in specific subgroups. For example, *BIRC3* was frequently altered in MMSET but rarely in other subgroups (14.6% of MMSET with alteration, 1.4% of others: *p-value* < 0.001; Table 3). In addition, *CDKN2C* and *RB1* were more frequently altered in the D2 and MMSET subgroups (*p*-values 0.019 and < 0.001, respectively). Mutations and deletions of *TRAF3*, a gene in the alternative NFκB pathway [15, 16], were enriched in high expressers of *CCND2* (9.2% of D2, MMSET, and MAF, 1.4% of D1-HRD and CCND1-11q13: *p*-value < 0.001). A full compendium of gene mutation and deletion counts both overall and split across TC-6 and UAMS molecular subgroups is available in Supplementary Table 2.

### Patterns of differential expression associated with RAS-RAF mutations

**Figure 2:**
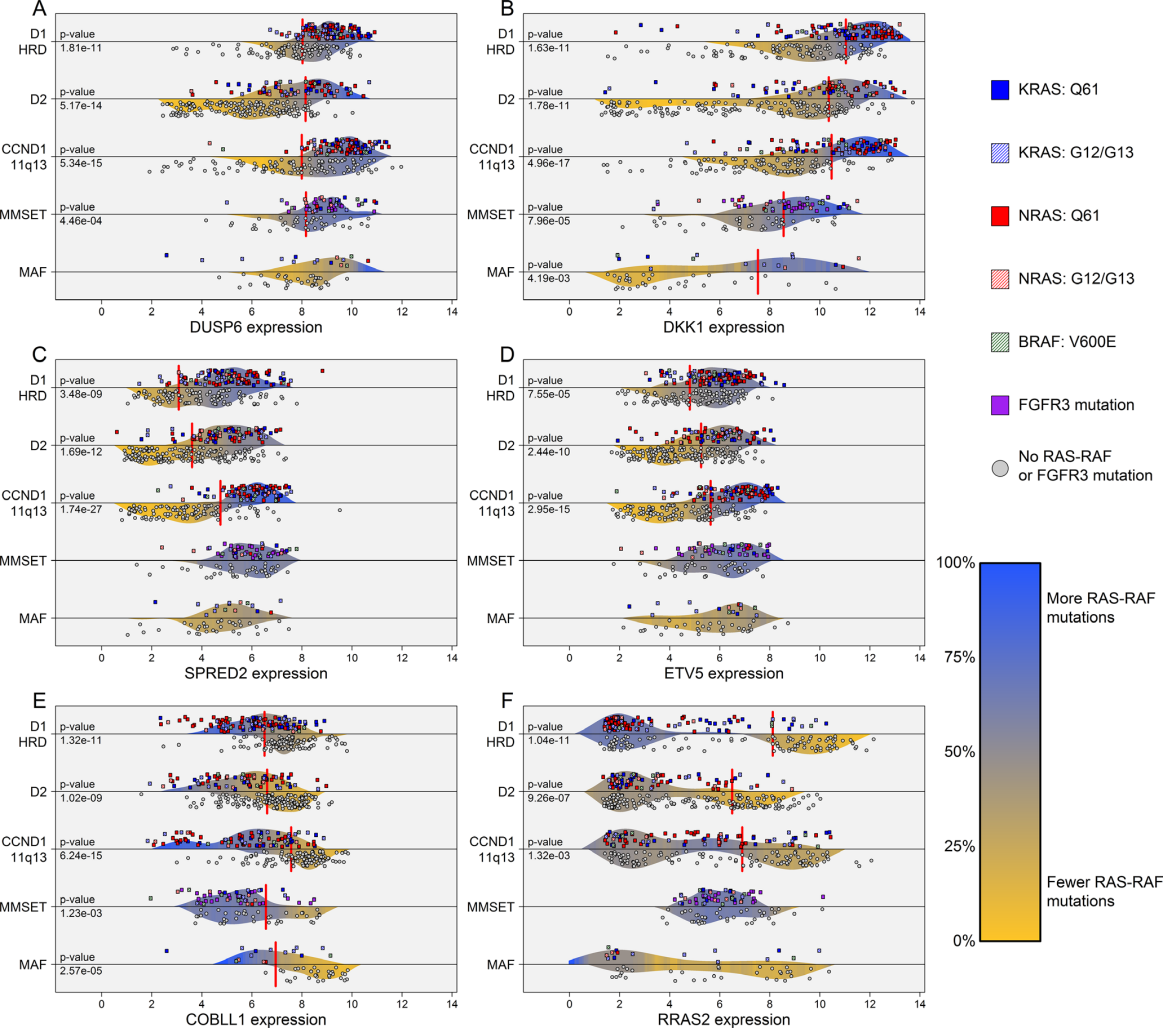
Differential gene expression patterns associated with presence of RAS-RAF mutations across TC-6 subgroups The gene expression levels of *DUSP6*, *DKK1*, *SPRED2*, *ETV5*, and *COBLL1* were highly associated with the presence of RAS-RAF or *FGFR3* mutations across all cases (**A–E**). *COBLL1* expression is negatively associated with presence of RAS-RAF mutation while remaining genes are positively associated. The RAS viral oncogene, *RRAS2*, has a dichotomous expression pattern in the D1-HRD subgroup where high expressers of *RRAS2* have few RAS-RAF mutations (**F**). Density curves are colored according to localized rate of RAS-RAF/FGFR3 mutation and represent probability distributions for mutated and non-mutated cases, separated by dividing horizontal lines. Vertical red lines indicate optimal thresholds of significance according to Fisher exact tests, limited to interior 60% of percentiles, where only highly significant *p*-values were reported (< 0.005). CCND3-6p21 subgroup not included due to sample size restraints (*n* =10). Gene expression represents log2 of GCRMA normalized data using the following probe or average of probes: *DUSP6* – 208891_at, 208892_s_at, 208893_s_at; *DKK1* – 204602_at; *SPRED2* – 212458_at; *ETV5* – 203349_s_at; *COBLL1* – 203641_s_at, 203642_s_at; and *RRAS2* – 212589_at, 212590_at.

Differential expression analysis revealed many genes to have patterns of expression highly associated with the presence of RAS-RAF and *FGFR3* mutations. Probes for *DUSP6*, *DKK1*, *SPRED2*, *COBLL1*, and *ETV5* were the most significantly associated with presence of RAS-RAF or *FGFR3* mutations across all cases (Supplementary Table 3 and Figure 2A–2E). Additional analyses revealed that for specific subgroups, certain genes were highly associated with the presence of RAS-RAF mutations. For example, sprouty-related protein gene *SPRED2*, established as a negative regulator of MAPK/ERK signaling [17, 18], was positively associated with the presence of RAS-RAF mutations, especially in the CCND1-11q13 subgroup. This differential expression pattern of *SPRED2* in CCND1-11q13 cases provides a nearly dichotomous split of RAS-RAF mutations at a certain threshold of *SPRED2* expression, i.e. 81.4% of cases with *SPRED2* expression above and 8.7% of cases with *SPRED2* expression below had RAS-RAF mutations (79/97 cases with *SPRED2* expression above 4.65 versus 9/104 below; *p*-value < 0.001; Figure 2C). In addition, the expression of the oncogene *RRAS2* has a dichotomous distribution across D1-HRD cases, i.e. differential clusters of low and high expressers, where high expression was negatively associated with presence of RAS-RAF mutations (17% of high *RRAS2* expressers with RAS-RAF mutations, 64% in low expressers; *p-value* < 0.001; Figure 2F). We note that these patterns of gene expression associated with RAS-RAF mutations are capable of further subtyping molecular subgroups of myeloma, especially in the D1-HRD, D2, and CCND1-11q13 subgroups where significant gene expression patterns were observed (Supplementary Figure 3).

We noted that two key genes observed to be significantly differentially expressed according to presence of RAS-RAF mutations were included in previous studies that defined the molecular subgroups of MM. Most notably, *DUSP6*, a negative regulator of MAPK/ERK signaling [19], is one of the top under-expressed genes in the LB (low bone) subgroup of the UAMS molecular subgroups and also under-expressed in the PRL3 subgroup of the HOVON model. We observed *DUSP6* expression to be positively associated with the presence of RAS-RAF mutation, thus these prior models of MM subtyping unknowingly identified cohorts that were negatively enriched for the presence of RAS-RAF mutations. We validated this by classifying our F1H cohort according to UAMS molecular subgroups and observed LB cases to have the lowest rate of RAS-RAF mutation (17.3%), while CD-1 (40.6%), HY (48.1%), and PR (43.8%) subgroups had significantly higher rates (LB RAS-RAF mutation rate vs other molecular subtypes: *p-value* = 0.001). In addition, the expression of *DKK1*, a known Wnt-signaling antagonist [20], was previously described as up-regulated in HY and down-regulated in MF subgroups in the UAMS molecular subgroups. We observed similar patterns of expression in D1-HRD and MAF subgroups, and its positive association with the presence of RAS-RAF mutations (Figure 2B). Overall, due to the abundance of RAS-RAF mutations and distinct associated patterns of gene expression, gene-expression based subtyping in MM is likely to incorporate these clear transcriptional relationships either knowingly or unknowingly.

### NFκB signaling inversely associated with RAS-RAF mutations

**Figure 3:**
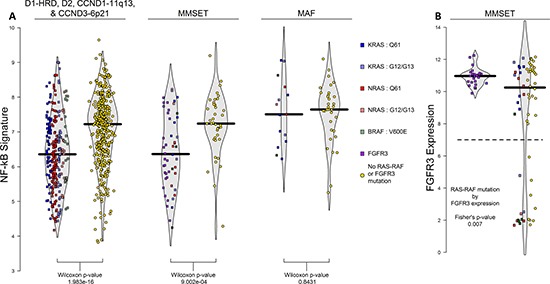
NFκB signature across TC-6 subgroups and *FGFR3* expression in MMSET subgroup The 11-gene NFκB signature is significantly associated with presence of RAS-RAF or *FGFR3* mutations in all TC-6 subgroups excluding MAF where cases without RAS-RAF mutations have higher levels of NFκB activation (**A**). When examining interaction of *FGFR3* expression and RAS-RAF mutation in MMSET, we observed that 56% (10/18) cases with low *FGFR3* expression have RAS-RAF mutations compared to 21% of remaining cases (15/71) (**B**).

NFκB signaling, according to the 11-gene NFκB signature [21], varied across the TC subgroups with significantly elevated levels in the MAF subgroup (*p*-values < 0.001 in TT and F1H; 0.17 in MRC-IX; Supplementary Figure 2B). Furthermore, NFκB signaling was negatively associated with presence of RAS-RAF and *FGFR3* mutations across all subgroups except for MAF (*p-value* < 0.001 for D1-HRD, D2, CCND1-11q13, and MMSET subgroups combined; *p-value* = 0.843 in MAF; Figure 3A).

### Unique mutational features of MMSET myeloma

MMSET cases are unique as they can overexpress and have activating mutations in *FGFR3*. Overall, 25 MMSET cases had *FGFR3* mutations (28.1%), 25 had a RAS-RAF mutation (28.1%), and 39 had neither (43.8%); co-occurrence of *FGFR3* and RAS-RAF mutations was not observed (Figure 1), consistent with both RAS-RAF and *FGFR3* mutations activating similar pathways and, therefore, being functionally redundant. The *FGFR3* locus is lost in 26% of MMSET cases [22], and consistent with this, we see lower expression of *FGFR3* in 20% (18 of 89) of MMSET cases in the F1H dataset. Over half of these cases (56%) had a RAS-RAF mutation, while only 21% of cases with high *FGFR3* expression had RAS-RAF mutations, indicating that *FGFR3* expression is more likely to be lost in the presence of a RAS-RAF mutation (*p-value* = 0.007; Figure 3B). In addition, both *FGFR3* and RAS-RAF mutated cases had lower NFκB signaling than those without either mutation (Figure 3A).

### Outcome of RAS-RAF codon mutations across subgroups

Despite a relatively short follow-up and heterogeneity in disease stage across our data set, we observed highly significant patterns in outcome associated with specific RAS-RAF codon mutations. Among subgroups with significant enrichment of Q61 *NRAS* mutations, we observed Q61 *NRAS* mutations to be associated with a favorable outcome in the t(11;14) subgroup, and a less favorable outcome in the D1-HRD and D2 subgroups (*p-value* = 0.001, independent of disease stage; Supplementary Figure 4).

For the MMSET subgroup, cases with RAS-RAF mutations had an inferior outcome compared to cases with *FGFR3* mutations or those without RAS-RAF mutations (*p-value* = 0.004, independent of disease stage). In addition, only 1/25 cases (4%) with an *FGFR3* mutation also had a *CDKN2C* or *RB1* alteration, while 28% of cases with RAS-RAF mutations had one or more of these alterations (*p-value* = 0.049). This is in keeping with alterations in *CDKN2C* and *RB1* being indicators of late stage disease [23, 24], thus the absence of these alterations in *FGFR3* mutated cases suggests *FGFR3* mutations as early events and RAS-RAF mutations as markers of late stage disease in t(4;14) MM.

### Alterations of *TP53*, *CDKN2C, and RB1* associated with adverse outcome

**Figure 4:**
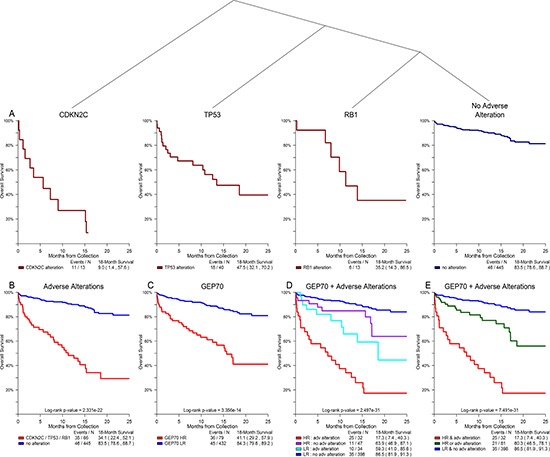
Alterations of *TP53, CDKN2C*, and *RB1* showed significant association with overall survival in non-relapse clinical setting Conditional inference tree analysis of all possible DNA alterations against outcome revealed that alterations in *TP53*, *CDKN2C*, and *RB1* were significantly associated with an adverse outcome for all cases with disease stages prior to relapse (**A**). The subset of cases with one or more of these adverse alterations had an 18-month OS rate that was statistically similar to that of GEP70 HR cases (**B, C**). Combining GEP70 risk and the presence of adverse alterations identified three cohorts with distinct clinical course: poor, intermediate, and standard outcomes (**D, E**).

A conditional inference tree analysis of all possible DNA variants against OS showed that alterations in *TP53*, *CDKN2C*, and *RB1* were significant prognostic factors across the F1H data set when excluding cases at or near relapse (Figure 4A). The subset of 66 non-relapse cases (12.9% of all non-relapse cases) with one or more of these adverse alterations had an extremely poor prognosis with an 18-month OS rate of 34.1%—statistically similar to the rate observed in GEP70 HR cases (*p-value* = 0.157; Figure 4B-4C). The combination of GEP70 risk and presence of adverse DNA alterations stratified all non-relapse cases into three arms with distinct clinical course: 32 cases (GEP70 HR and any adverse alteration) with an extremely poor outcome (17.3% OS at 18-months), 81 cases (GEP70 HR or any adverse alteration) with an intermediate outcome (60.3% OS rate at 18-months), and 398 cases (no adverse alterations and GEP70 LR) with a standard outcome (86.5% OS at 18-months) (*p-value* < 0.001; Figure 4D). Overall, this subset of alterations (*TP53*, *CDKN2C*, and *RB1*) is significantly associated with progressive disease stage where 25.9% of cases at or near relapse had one or more of these alterations compared to 12.9% in prior disease stages (*p-value* < 0.001, Table 2).

**Figure 5:**
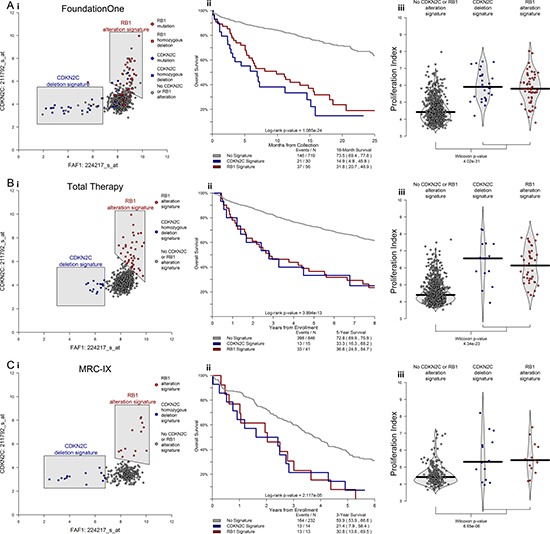
*CDKN2C* and *RB1* alterations elicit distinct patterns of gene expression that have significant impact on proliferation and clinical outcome in newly diagnosed MM Differential expression analysis revealed highly significant patterns of expression associated with alteration in *CDKN2C* and *RB1*. Namely that cases with deletion of *CDKN2C* have lower expression of *FAF1* and that cases with alterations of *RB1* have higher expression of *CDKN2C* (**Ai**). Cases with either of these distinct expression patterns had inferior outcomes (Aii) and higher 50-gene proliferation scores (Aiii). Identical patterns of inferior outcome and elevated proliferation were observed in both the Total Therapy and MRC-IX data sets of NDMM (**B, C**). In total, alterations in *CDKN2C* and *RB1* offer mutually exclusive paths that culminate in similar high-risk behavior.

In order to define a mechanistic basis for HR, we examined patterns of gene expression associated with these adverse alterations. We observed strong associations between *CDKN2C* deletions and low expression of *FAF1*, and that high expression of *CDKN2C* was associated with alterations of *RB1*. We also found cases exhibiting these expression patterns across two NDMM data sets, and observed significant associations with proliferation and outcome for cases exhibiting either expression pattern. Cases with either a low *FAF1* expression signature (associated with *CDKN2C* deletion) or high *CDKN2C* expression signature (associated with *RB1* alteration) had an 18-month OS rate of 26.9% compared to 73.5% in cases without either signature (*p-value* < 0.001; Figure 5Aii). In addition, cases with either adverse expression signature had elevated proliferation indexes, according to the 50-gene proliferation signature [25] (*p-value* < 0.001; Figure 5Aiii). These patterns of gene expression, outcome, and proliferation were validated in both the TT and MRC-IX datasets of NDMM cases (Figure 5B–5C).

We note that within the F1H data set, *CDKN2C* alterations (29 cases) and *RB1* alterations (38 cases) did not co-occur in any single case. This observation, along with the disparate patterns of gene expression, is consistent with alterations in *CDKN2C* and *RB1* offering mutually exclusive paths to elevated proliferation and high-risk behavior.

## DISCUSSION

We show for the first time a clear difference in mutational spectrum across the molecular subgroups of MM defined by an updated TC algorithm. Specifically, we show that *NRAS* mutations at Q61 are common in HRD and t(11;14) myeloma but rare in MMSET and MAF. This heterogeneity in RAS codon mutations parallels the diversity in primary translocation and cyclin D initiating events in myeloma. These primary events yield diverse genetic backgrounds that likely influence the rate, type, and impact of the acquisition of secondary mutations and deletions. Overall, this analysis aims to describe the unique distribution and clinical impact of key mutations across the molecular subtypes of myeloma while highlighting the importance of defining RAS mutations at the codon level.

Both primary TC event and RAS-RAF mutation have distinct resultant patterns in gene expression. We used the gene expression patterns associated with cyclin D and translocation events to generate an updated model that defines all cases according to six primary TC events. This model is unique from prior TC models in that it uses additional secondary genes, e.g. *SLC8A1* up-regulation in t(11;14) or *DSG2* up-regulation in t(4;14) myeloma, incorporated into a support vector machine (SVM) classification model to determine optimal subgroups in a simultaneous rather than dichotomous step-wise fashion. This model is distinct from the molecular subtyping models because it groups cytogenetically similar cases together rather than allowing secondary events to determine subgroup, e.g. cases with t(4;14) and t(11;14) may classify as PR (proliferative) in UAMS molecular subgroups. We also observed strong patterns of gene expression associated with the presence of RAS-RAF mutations. Most notably, *DKK1*, *DUSP6*, and *SPRED2* were positively associated with the presence of RAS-RAF mutations. This pattern was strongest in subgroups with higher rates of *NRAS* mutations at Q61, i.e. D1-HRD, D2, and CCND1-11q13. We suspect that the increased expression of MAPK/ERK antagonists (*DUSP6* and *SPRED2*) associated with Q61 *NRAS* mutations is a result of a regulating transcriptional response attempting to counteract activating RAS mutations, i.e. a repeating negative feedback mechanism.

We show that all non-MAF cases lacking a RAS-RAF or *FGFR3* mutation have increased NFκB signaling. This reciprocal relationship between NFκB signaling and activating RAS-RAF mutations is consistent with a functional similarity of signaling via these pathways and, therefore, functional redundancy. We propose, based on this observation, that MM is characterized by growth signaling delivered primarily via MAPK or NFκB pathways in a mutually exclusive fashion. The MAF subgroup is unique in this respect as all cases have elevated NFκB signaling irrespective of their RAS mutational status, likely indicative of their unique genetic background [26].

DNA alterations of *TP53*, *CDKN2C*, and *RB1* were identified as key markers of progressive disease and associated with adverse outcome in a non-relapse MM setting. We also presented the unique interaction between deletion of *CDKN2C* and alteration of *RB1* where both were mutually exclusive with distinct downstream signals in gene expression, yet each impart similar paths to elevated proliferation and adverse outcome. As both *CDKN2C* and *RB1* interact with G1/S cell cycle checkpoint through *CDK4/6* [27–29], they are potentially targetable through CDK4/6 inhibition [30], for which it has been shown in other cancers that co-deletion of *CDKN2C* and *CDKN2A* with functional presence of *RB1* increases sensitivity in cell lines [31]. A better understanding of the interaction of these two key prognostic markers in MM could open new possibilities for targeted therapy. Overall, this work aims to serve as a fundamental step in the transition from panel-based DNA assessment and GEP-based expression analysis to RNA-Seq and whole genome assessments that fully examine the complete mutational landscape of MM.

## MATERIALS AND METHODS

A set of 805 UAMS GEP samples underwent targeted sequencing using the FoundationOne Heme (F1H) mutational assay (Foundation Medicine, Cambridge, MA) annotated for known and likely mutations and deletions with variant allele frequencies at or above five percent. Additionally, *KRAS*, *NRAS*, and *BRAF* mutations were limited to missense mutations annotated for known activating mutations: codons G12, G13, and Q61 for *K* and *N RAS* and V600E for *BRAF*. Additional mutations in non RAS-RAF genes included all varieties of short variants: frameshift, missense, nonsense, etc. This series has a median follow-up of 13.5 months with samples collected at various disease stages including 23% prior to treatment, 41% in treatment, and 36% at or near relapse (within 90 days +/− of progression event). This data set was analyzed for associations in mutation and deletion associated with TC subgroups.

An additional data set of 902 UAMS Myeloma Institute gene expression profiling (GEP) samples from newly diagnosed (NDMM) patients accrued to Total Therapy (TT) trials between 2000 and 2010 had a median follow-up of over 10 years and was used to develop an updated TC classification model. iFISH data for 1q+, 1p–, 13q–, and 17p– gathered at baseline were available but not in full. Patients gave written informed consent for bone marrow sampling and the research was approved by the institutional review board of UAMS. This data set of 902 NDMM cases was used to train the updated TC algorithm.

For all GEP data, plasma cells were CD138-purified from bone marrow aspirates and processed on U133 Plus 2.0 microarrays (Affymetrix, Santa Clara, CA) [32]. CEL files were normalized using GCRMA [33] for application of updated TC algorithm. MAS5 normalization was also performed when necessary, e.g. for calculation of GEP70 and UAMS molecular subtypes. All expression data was normalized using *R* Bioconductor and transformed to the UAMS TT2 and TT3 NDMM standard according to a variant of M-ComBat [34].

### Updated TC classifier

An updated GEP-based TC classifier was developed (TC-6) which reflects the primary molecular events in MM. The model was trained on the NDMM TT set of 902 cases using a support vector machine (SVM) [35] where translocation groups were identified according to clear gene expression spikes with remaining non-translocated cases classified according to cyclin-D dysregulation. The model uses 24 probes chosen for their power to discriminate translocation and cyclin-D dysregulated subgroups and is available online at http://github.com/SteinCK/TC-6 for public use.

We have simplified the original eight to six primary subgroups, classifying non-translocated MM into two groups according to deregulation of D-group cyclin (D1 or D2) without a hybrid class with expression in both, i.e. D1 + D2. This reflects our inability to elicit a cluster with expression in both *CCND1* and *CCND2* that is a distinct entity apart from the D1 and D2 subgroups. Additional analysis of NGS copy number data also supports the existence of a singular D1-HRD subgroup, where a homogeneous cohort with copy number gains of chromosomes 5, 9, 15, 19, and, uniquely, 11, emerges that also identifies as D1-HRD according to TC-6 classification [36].

The methods used to perform subgroup determinations were also updated considerably from original TC methodology in that determinations are now performed simultaneously with an advanced classification system (SVM) rather than in a dichotomous step-wise fashion using simple binary thresholds. This allows for more sophisticated discrimination for each individual case that weighs the entire composition of gene expression to form an optimal subgroup determination rather than individual probes with binary outcomes. We also address sample purity and contamination concerns in our methodology by including healthy, normal plasma cell (NPC) sample controls as a distinct subgroup in model training. Thus across our heterogeneous data set we limited our analyses to samples that failed to cluster with the NPC contamination cluster. In addition, we built this classifier on GCRMA rather than MAS5 normalized expression data as GCRMA normalization has been shown to improve overall accuracy in comparative studies [37, 38]. This updated TC-6 model validated well on an external set of 259 GEP samples from the MRC-IX trial [39, 40], where it accurately predicted iFISH translocation designations (Supplementary Table 1).

All statistical analyses were performed in *R* with primary use of the *survival* [41], *party* [42], *glmnet* [43], and *e1071* [44] packages. Differential expression analysis of associations between mutation and GEP data was performed with *limma* [45]. All *p*-values reported for contingency tables, continuous variable, and survival comparisons were determined by Fisher's exact tests, Wilcoxon signed rank tests, and log-rank tests, respectively.

## SUPPLEMENTARY MATERIALS FIGURES AND TABLES






