# Supplementary material for: The varied distribution and impact of RAS codon and other key DNA alterations across the translocation cyclin D subgroups in multiple myeloma
Source: Oncotarget. 2017 Feb 24;8(17):27854–67. doi: 10.18632/oncotarget.15718 (PMC5438613; doi:10.18632/oncotarget.15718)
Supplement: Supplementary file 1 [file oncotarget-08-27854-s001.pdf]

The varied distribution and impact of RAS codon and other key DNA alterations across the translocation cyclin D subgroups in multiple myeloma

Supplementary Materials

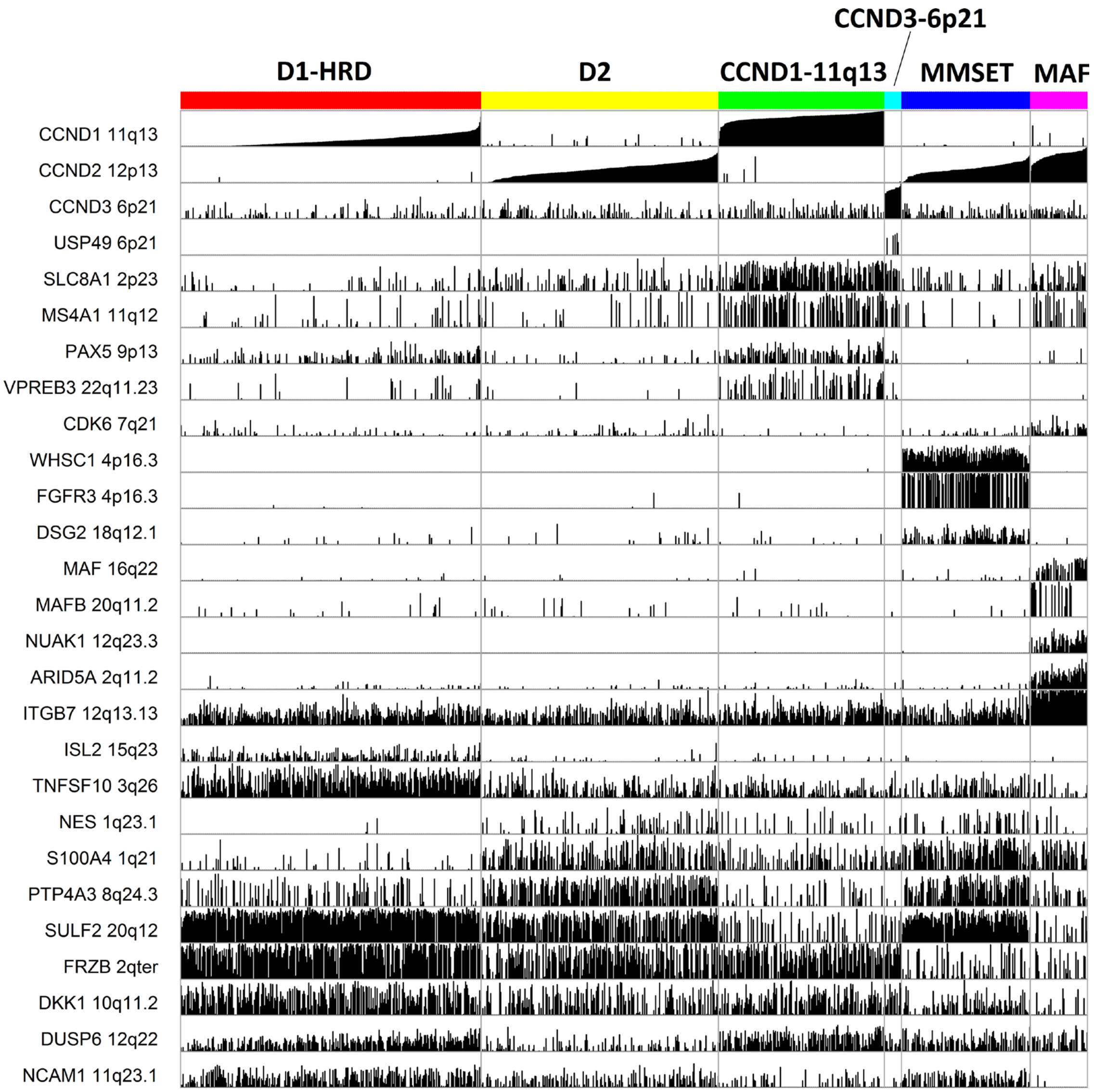

Supplementary Figure 1: Ordered bar plot of key gene expression associations across TC-6 subgroups. Gene expression features of TC-6 subgroups are distinct. Each subgroup has a primary dysregulated D-group cyclin and/or translocation genes and secondary genes that help define its members, e.g. CCND1-11q13 cases overexpress *CCND1* (primary) and *SLC8A1* (secondary) while MMSET cases overexpress *WHSC1* (or *MMSET*), *CCND2* and *DSG2* (secondary). The D1-HRD subgroup has intermediate expression of *CCND1* with elevated expression of hyperdiploid-related genes located on odd-numbered chromosomes (*ISL2*, *TNFSF10*). The D2 subgroup has high *CCND2* and *PTP4A3* expression without an *IGH* translocation. The CCND1-11q13 subgroup is composed of cases with t(11;14) *IGH* translocations, elevated expression of *CCND1* and *SLC8A1*, and over expression of the *CD20* (*MS4A1*) in 54% of cases. CCND3-6p21 is a rare subtype with spiked expression of *CCND3* often accompanied by high *USP49* expression. The MMSET subgroup includes all cases with 4p16 *IGH* translocations, elevated expression of *MMSET* (*WHSC1*), with or without high expression of *FGFR3*. The MAF subgroup is composed of cases of all three subtypes of MAF-associated *IGH* translocations: 16q23 (c-MAF), 20q12 (MAFB), and 8q24 (MAFA, rare). These subtypes have elevated expression of MAF-associated genes such as *NUAK1*, *ITGB7*, and *ARID5A* with spiked expression in *MAF*, *MAFB*, or *MAFA*, respectively. We note that RNA-Seq analysis offers confirmation of 8q24-MAFA cases as probes for MAFA transcripts are available. Log2 expression values for key genes are shown, sorted by cyclin D expression. Expression values shown range from a minimum raw value of 50 and to a maximum of 7,500.

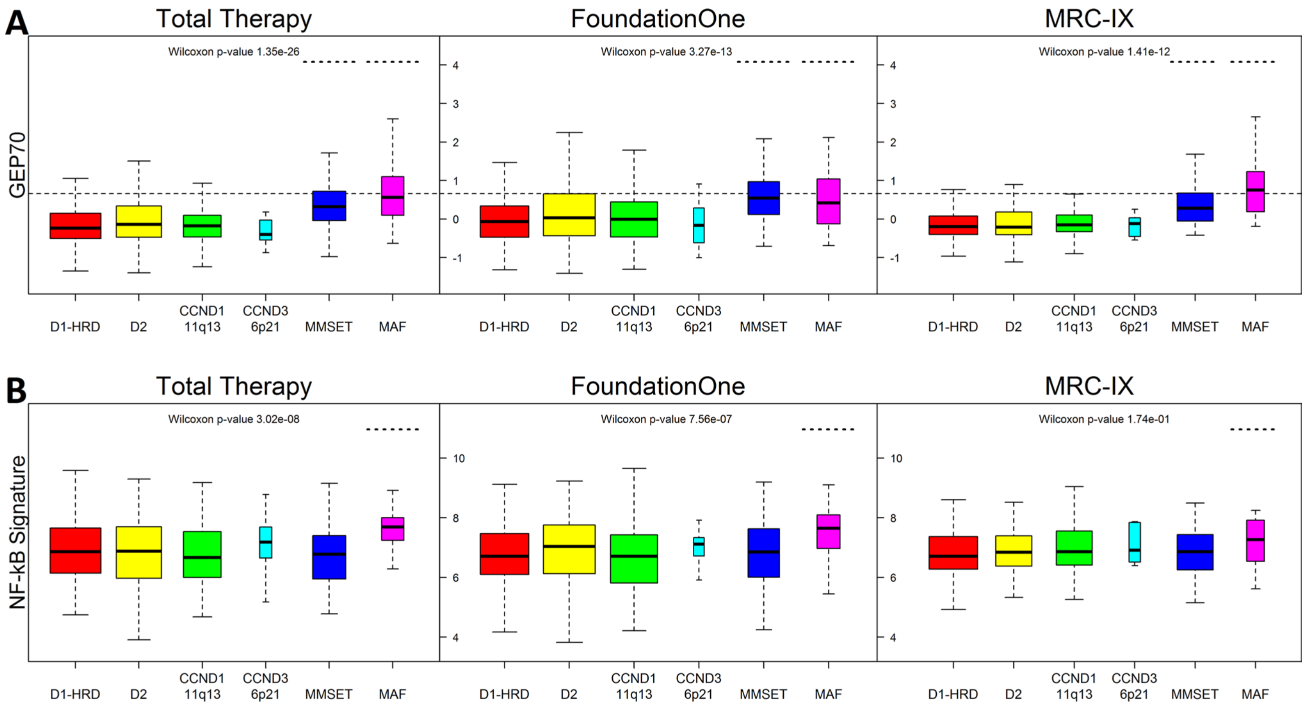

Supplementary Figure 2: GEP70 and NFkB signature values across TC-6 subgroups. TC-6 subgroups have varied gene expression scores for GEP70 and NFkB signature. GEP70 scores are elevated significantly in the MMSET and MAF subgroups across all three data sets (*p*-values < 0.001) (**A**). NFkB signature is highly variable, however it is uniquely elevated in the MAF subgroup (*p*-values < 0.001 in TT and F1H; *p*-value = 0.17 in MRC-IX) (**B**).

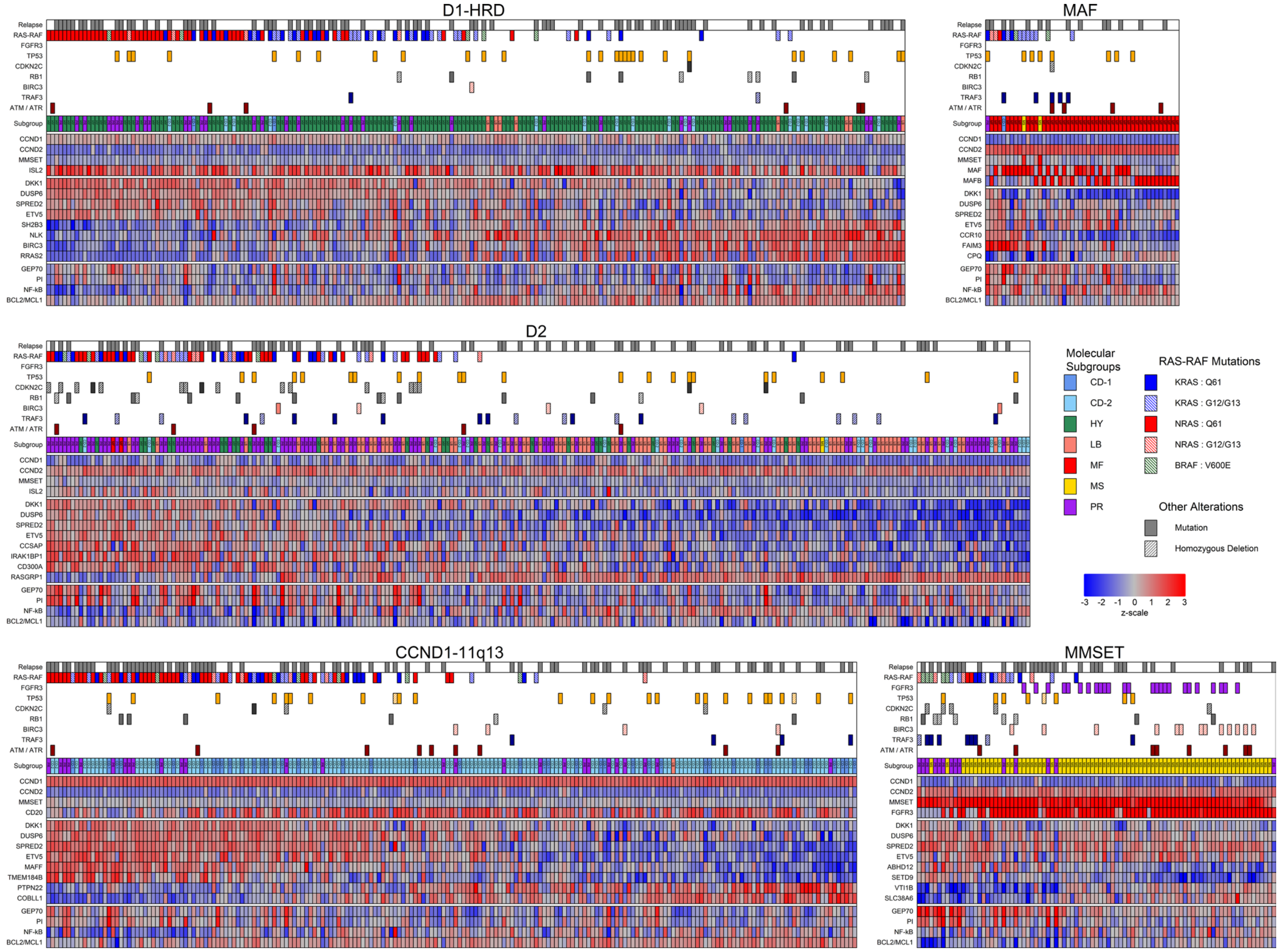

Supplementary Figure 3: Gene expression heat map and block plot of key alterations in TC-6 subgroups. Strong associations between gene expression and presence of RAS-RAF mutations exist across the TC-6 subgroups. In particular, the D1-HRD, D2, and CCND1-11q13 subgroups have a significant association where high expression of *DKK1*, *DUSP6*, *SPRED2*, and *ETV5* is observed in the presence of RAS-RAF activating mutations. Additional genes with significant association with RAS-RAF mutations were included in each subgroup heatmap, e.g. the expression of *BIRC3* and *RRAS2* are significantly negatively associated with presence of RAS-RAF activating mutations in the D1-HRD subgroup. The top section of each panel shows a block plot of key alterations across the TC-6 subgroups shaded according to legend. Heatmap of up and down-regulated genes, colored in red and blue, includes additional genes for each subgroup selected systematically according to regularized regression (lasso). Cases are sorted from left to right according to likelihood of RAS-RAF mutation as predicted from GEP-based subgroup signatures.

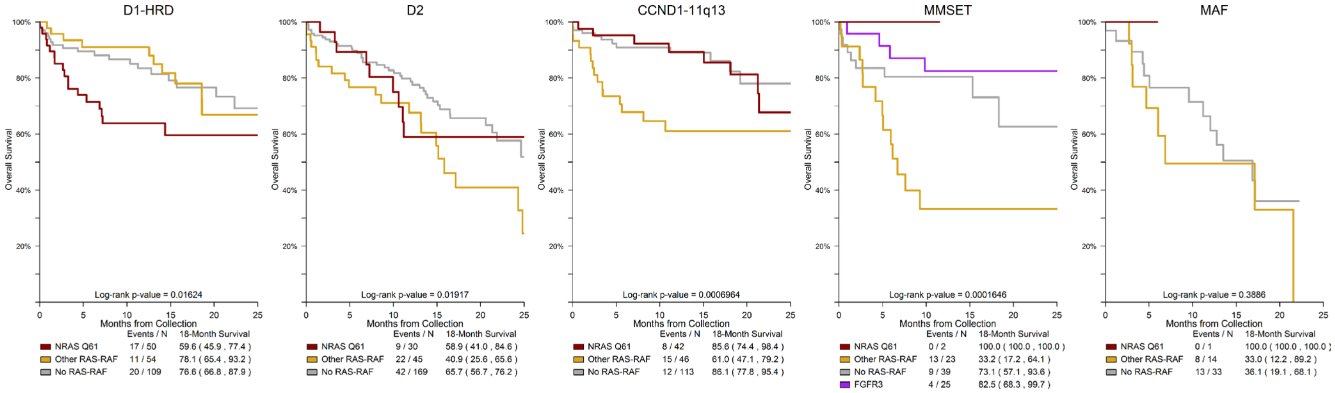

Supplementary Figure 4: Kaplan-Meier overall survival curves for RAS-RAF and *FGFR3* mutations by TC-6 subgroups. We observe that in the subgroups that are enriched for *NRAS* mutations at Q61, mutations of *NRAS* at Q61 impart a less favorable outcome in CCND1-11q13 subgroup than in D1-HRD and D2 subgroups. Furthermore, RAS-RAF mutations in MMSET myeloma have less favorable outcome than cases with *FGFR3* mutations or without RAS-RAF mutations.

Supplementary Table 1: Validation results of TC-6 model in external MRC-IX data

		t(4;14)	t(11;14)	t(14;16) or t(14;20)
**MRC-IX**	Accuracy	99.2	98.3	97.9
	Sensitivity	97.7<br>(86.5, 99.9)	97.8<br>(87.0, 99.9)	87.5<br>(46.7, 99.3)
	Specificity	99.5<br>(96.7, 100.0)	98.5<br>(95.2, 99.6)	98.3<br>(95.4, 99.4)

iFISH defined translocations from the MRC-IX data and GEP-predicted translocations from TC-6 model showed strong agreement. Identification of t(4;14) translocations by iFISH had 99% agreement with the TC-6 GEP-based prediction of MMSET subtype, 98.3% agreement between t(11;14) and CCND1-11q13 subtype, and 97.9% agreement between t(14;16) or t(14;20) identification and MAF subtype.

Supplementary Table 2: Compendium of mutation and deletion counts overall and split by TC-6 and UAMS molecular subgroups. See Supplementary_Table_2

Supplementary Table 3: Differentially expressed genes associated with presence of RAS-RAF and *FGFR3* mutations. See Supplementary_Table_3
