# Supplementary material for: The varied distribution and impact of RAS codon and other key DNA alterations across the translocation cyclin D subgroups in multiple myeloma
Source: Oncotarget. 2017 Feb 24;8(17):27854–67. doi: 10.18632/oncotarget.15718 (PMC5438613; doi:10.18632/oncotarget.15718)
Supplement: Supplementary file 3 [file oncotarget-08-27854-s003.docx]

Supplementary Table 3. Differentially expressed genes associated with presence of RAS-RAF and *FGFR3* mutations.

Differential expression analysis was performed with *limma* across all cases and found the following probes to be significantly associated with presence of RAS-RAF mutations. Probes analyzed were limited to those with standard deviation above 1, expression above log2( 20 ), and log2 fold changes above 1.0. The top 100 significant probes are listed below.

| ProbeID | Gene | Chr Location | Ave Expression | logFC | p-value | q-value |
| --- | --- | --- | --- | --- | --- | --- |
| 212458_at | SPRED2 | chr2p14 | 4.43 | 1.65 | 1.54E-45 | 4.80E-42 |
| 208893_s_at | DUSP6 | chr12q22-q23 | 6.67 | 1.76 | 7.76E-43 | 1.21E-39 |
| 208892_s_at | DUSP6 | chr12q22-q23 | 8.21 | 1.95 | 1.86E-39 | 1.94E-36 |
| 208891_at | DUSP6 | chr12q22-q23 | 8.45 | 1.90 | 2.70E-39 | 2.11E-36 |
| 203641_s_at | COBLL1 | chr2q24.3 | 4.48 | -1.41 | 4.01E-37 | 2.51E-34 |
| 204602_at | DKK1 | chr10q11.2 | 8.51 | 2.67 | 4.84E-37 | 2.52E-34 |
| 203349_s_at | ETV5 | chr3q28 | 5.28 | 1.29 | 1.15E-33 | 5.12E-31 |
| 203642_s_at | COBLL1 | chr2q24.3 | 8.79 | -1.45 | 2.43E-33 | 9.48E-31 |
| 220850_at | MORC1 | chr3q13 | 5.30 | 2.33 | 3.87E-33 | 1.34E-30 |
| 205590_at | RASGRP1 | chr15q14 | 6.14 | -2.26 | 4.43E-27 | 1.26E-24 |
| 226837_at | SPRED1 | chr15q14 | 5.80 | 1.71 | 2.26E-26 | 5.88E-24 |
| 213793_s_at | HOMER1 | chr5q14.2 | 5.50 | 1.73 | 6.43E-25 | 1.54E-22 |
| 201059_at | CTTN | chr11q13 | 5.45 | -1.13 | 3.22E-23 | 7.17E-21 |
| 226651_at | HOMER1 | chr5q14.2 | 5.94 | 1.28 | 2.21E-22 | 4.32E-20 |
| 215967_s_at | LY9 | chr1q23.3 | 6.61 | 1.00 | 6.10E-22 | 1.06E-19 |
| 209201_x_at | CXCR4 | chr2q21 | 10.02 | -1.38 | 1.27E-21 | 1.98E-19 |
| 219743_at | HEY2 | chr6q21 | 5.92 | 1.17 | 1.60E-21 | 2.27E-19 |
| 211919_s_at | CXCR4 | chr2q21 | 9.89 | -1.32 | 2.60E-21 | 3.53E-19 |
| 230499_at | BIRC3 | chr11q22 | 5.27 | -1.35 | 3.63E-21 | 4.72E-19 |
| 240890_at | LOC643733 | chr11q22.3 | 6.06 | 1.47 | 7.18E-21 | 8.97E-19 |
| 204588_s_at | SLC7A7 | chr14q11.2 | 6.65 | -1.00 | 1.82E-20 | 2.10E-18 |
| 226247_at | PLEKHA1 | chr10q26.13 | 4.49 | -1.42 | 2.35E-20 | 2.62E-18 |
| 222921_s_at | HEY2 | chr6q21 | 6.79 | 1.06 | 5.88E-20 | 6.33E-18 |
| 213355_at | ST3GAL6 | chr3q12.1 | 10.07 | 1.09 | 5.27E-19 | 4.70E-17 |
| 210942_s_at | ST3GAL6 | chr3q12.1 | 8.73 | 1.10 | 5.63E-19 | 4.75E-17 |
| 36711_at | MAFF | chr22q13.1 | 6.81 | 1.01 | 1.33E-18 | 9.81E-17 |
| 228377_at | KLHL14 | chr18q12.1 | 6.16 | -2.14 | 2.67E-18 | 1.81E-16 |
| 222783_s_at | SMOC1 | chr14q24.2 | 6.42 | 1.34 | 3.65E-18 | 2.37E-16 |
| 203998_s_at | SYT1 | chr12cen-q21 | 6.30 | 1.55 | 5.01E-18 | 3.13E-16 |
| 237515_at | TMEM56 | chr1p21.3 | 6.37 | -1.01 | 1.11E-17 | 6.64E-16 |
| 201952_at | ALCAM | chr3q13.1 | 8.64 | -1.01 | 4.06E-17 | 2.30E-15 |
| 205923_at | RELN | chr7q22 | 5.74 | 1.72 | 6.47E-17 | 3.54E-15 |
| 203999_at | SYT1 | chr12cen-q21 | 6.14 | 1.32 | 1.36E-16 | 7.07E-15 |
| 1557765_at | LOC643401 | chr5p14.1 | 5.50 | 1.32 | 5.21E-16 | 2.47E-14 |
| 228284_at | TLE1 | chr9q21.32 | 6.63 | -1.49 | 7.50E-16 | 3.39E-14 |
| 210538_s_at | BIRC3 | chr11q22 | 9.61 | -1.43 | 8.64E-16 | 3.86E-14 |
| 203471_s_at | PLEK | chr2p13.3 | 6.34 | -1.04 | 3.38E-15 | 1.35E-13 |
| 205098_at | CCR1 | chr3p21 | 6.06 | 1.47 | 4.91E-15 | 1.92E-13 |
| 205099_s_at | CCR1 | chr3p21 | 4.95 | 1.03 | 1.23E-14 | 4.51E-13 |
| 1555638_a_at | SAMSN1 | chr21q11 | 7.63 | -1.14 | 2.19E-14 | 7.50E-13 |
| 241844_x_at | TMEM156 | chr4p14 | 6.94 | -1.29 | 2.59E-14 | 8.69E-13 |
| 220565_at | CCR10 | chr17q21.1-q21.3 | 5.91 | 1.22 | 3.09E-14 | 9.95E-13 |
| 220169_at | TMEM156 | chr4p14 | 6.16 | -1.17 | 3.58E-14 | 1.13E-12 |
| 211986_at | AHNAK | chr11q12.2 | 7.17 | -1.05 | 3.61E-14 | 1.13E-12 |
| 34210_at | CD52 | chr1p36 | 4.91 | -1.26 | 4.23E-14 | 1.26E-12 |
| 206150_at | CD27 | chr12p13 | 6.98 | -1.28 | 6.61E-14 | 1.91E-12 |
| 203221_at | TLE1 | chr9q21.32 | 6.55 | -1.21 | 1.36E-13 | 3.70E-12 |
| 221911_at | ETV1 | chr7p21.3 | 4.75 | 1.78 | 1.38E-13 | 3.70E-12 |
| 204014_at | DUSP4 | chr8p12-p11 | 5.55 | 1.39 | 1.71E-13 | 4.49E-12 |
| 209933_s_at | CD300A | chr17q25.1 | 5.14 | 1.04 | 2.26E-13 | 5.78E-12 |
| 208456_s_at | RRAS2 | chr11p15.2 | 4.66 | -1.29 | 3.81E-13 | 9.45E-12 |
| 228245_s_at | LOC100509445 | chr12p11.21 | 7.07 | 1.05 | 4.11E-13 | 1.01E-11 |
| 227404_s_at | EGR1 | chr5q31.1 | 5.28 | 1.08 | 4.57E-13 | 1.11E-11 |
| 204661_at | CD52 | chr1p36 | 5.92 | -1.04 | 6.38E-13 | 1.51E-11 |
| 209708_at | MOXD1 | chr6q23.2 | 7.11 | 1.24 | 6.92E-13 | 1.63E-11 |
| 201693_s_at | EGR1 | chr5q31.1 | 4.60 | 1.15 | 7.58E-13 | 1.76E-11 |
| 215789_s_at | AJAP1 | chr1p36.32 | 4.37 | 1.19 | 8.21E-13 | 1.87E-11 |
| 1554474_a_at | MOXD1 | chr6q23.2 | 6.97 | 1.06 | 1.05E-12 | 2.35E-11 |
| 212590_at | RRAS2 | chr11p15.2 | 4.99 | -1.37 | 1.45E-12 | 3.20E-11 |
| 202411_at | IFI27 | chr14q32 | 6.26 | 1.63 | 5.31E-12 | 1.04E-10 |
| 242517_at | KISS1R | chr19p13.3 | 4.35 | -1.04 | 7.45E-12 | 1.40E-10 |
| 242100_at | CHSY3 | chr5q23.3 | 6.44 | 1.20 | 9.22E-12 | 1.70E-10 |
| 226702_at | CMPK2 | chr2p25.2 | 7.09 | 1.22 | 1.21E-11 | 2.19E-10 |
| 214070_s_at | ATP10B | chr5q34 | 5.83 | 1.56 | 1.23E-11 | 2.21E-10 |
| 235763_at | SLC44A5 | chr1p31.1 | 4.52 | 1.16 | 2.14E-11 | 3.66E-10 |
| 229070_at | ADTRP | chr6p24.1 | 6.62 | -1.40 | 2.30E-11 | 3.91E-10 |
| 226682_at | RORA | chr15q22.2 | 7.09 | -1.04 | 3.43E-11 | 5.61E-10 |
| 201694_s_at | EGR1 | chr5q31.1 | 7.61 | 1.28 | 3.53E-11 | 5.74E-10 |
| 220330_s_at | SAMSN1 | chr21q11 | 9.09 | -1.10 | 7.52E-11 | 1.18E-09 |
| 218678_at | NES | chr1q23.1 | 4.43 | 1.20 | 7.70E-11 | 1.20E-09 |
| 204944_at | PTPRG | chr3p21-p14 | 6.07 | 1.03 | 8.97E-11 | 1.37E-09 |
| 212589_at | RRAS2 | chr11p15.2 | 5.37 | -1.43 | 9.75E-11 | 1.47E-09 |
| 203989_x_at | F2R | chr5q13 | 5.04 | 1.22 | 1.46E-10 | 2.15E-09 |
| 210432_s_at | SCN3A | chr2q24 | 4.80 | 1.41 | 4.64E-10 | 6.25E-09 |
| 229435_at | GLIS3 | chr9p24.2 | 4.48 | 1.05 | 5.87E-10 | 7.68E-09 |
| 229437_at | MIR155 | chr21q21.3 | 7.03 | -1.22 | 5.88E-10 | 7.68E-09 |
| 235048_at | FAM169A | chr5q13.3 | 4.36 | 1.02 | 1.06E-09 | 1.34E-08 |
| 210715_s_at | SPINT2 | chr19q13.1 | 6.66 | -1.18 | 1.11E-09 | 1.39E-08 |
| 242625_at | RSAD2 | chr2p25.2 | 5.55 | 1.11 | 1.89E-09 | 2.27E-08 |
| 231807_at | KIAA1217 | chr10p12.31 | 6.86 | 1.03 | 2.64E-09 | 3.10E-08 |
| 201666_at | TIMP1 | chrXp11.3-p11.23 | 7.61 | 1.24 | 9.55E-09 | 1.01E-07 |
| 217258_x_at | IGLV1-44 | chr22q11.2 | 5.75 | -1.26 | 1.18E-08 | 1.21E-07 |
| 227394_at | NCAM1 | chr11q23.1 | 6.19 | 1.17 | 1.30E-08 | 1.33E-07 |
| 217227_x_at | IGLV1-44 | chr22q11.2 | 6.75 | -1.16 | 1.32E-08 | 1.34E-07 |
| 219463_at | LAMP5 | chr20p12 | 7.53 | 1.55 | 1.66E-08 | 1.65E-07 |
| 212843_at | NCAM1 | chr11q23.1 | 6.84 | 1.32 | 1.70E-08 | 1.68E-07 |
| 239481_at | FAM133A | chrXq21.32 | 8.14 | 1.06 | 3.42E-08 | 3.16E-07 |
| 202912_at | ADM | chr11p15.4 | 8.92 | 1.02 | 3.92E-08 | 3.57E-07 |
| 214973_x_at | IGHD | chr14q32.33 | 7.33 | -1.18 | 4.92E-08 | 4.37E-07 |
| 242881_x_at | LOC100506303 | chr14q11.1 | 6.34 | 1.13 | 5.06E-08 | 4.45E-07 |
| 212190_at | SERPINE2 | chr2q36.1 | 4.36 | 1.25 | 7.38E-08 | 6.18E-07 |
| 231131_at | FAM133A | chrXq21.32 | 7.37 | 1.13 | 9.13E-08 | 7.46E-07 |
| 224009_x_at | DHRS9 | chr2q31.1 | 5.44 | -1.19 | 1.06E-07 | 8.54E-07 |
| 213674_x_at | IGHD | chr14q32.33 | 6.74 | -1.34 | 1.72E-07 | 1.33E-06 |
| 224342_x_at | LOC96610 | chr22q11.22 | 6.94 | -1.20 | 2.19E-07 | 1.62E-06 |
| 215214_at | IGLC1 | chr22q11.2 | 7.59 | -1.07 | 2.45E-07 | 1.79E-06 |
| 209942_x_at | MAGEA3 | chrXq28 | 4.67 | 1.29 | 2.70E-07 | 1.94E-06 |
| 214612_x_at | MAGEA6 | chrXq28 | 5.17 | 1.27 | 2.82E-07 | 2.01E-06 |
| 223952_x_at | DHRS9 | chr2q31.1 | 5.52 | -1.13 | 3.24E-07 | 2.26E-06 |
| 217235_x_at | IGLL5 | chr22q11.22 | 8.19 | -1.06 | 7.43E-07 | 4.69E-06 |
